# Search for rare protein altering variants influencing susceptibility to multiple myeloma

**DOI:** 10.18632/oncotarget.15874

**Published:** 2017-03-03

**Authors:** Matthew Scales, Daniel Chubb, Sara E. Dobbins, David C. Johnson, Ni Li, Michael J. Sternberg, Neils Weinhold, Caleb Stein, Graham Jackson, Faith E. Davies, Brian A. Walker, Christopher P. Wardell, Richard S. Houlston, Gareth J. Morgan

**Affiliations:** ^1^ Division of Genetics and Epidemiology, The Institute of Cancer Research, Sutton, Surrey, UK; ^2^ Centre of Bioinformatics and Systems Biology, Department of Life Sciences, Imperial College London, London, UK; ^3^ Division of Molecular Pathology, The Institute of Cancer Research, Sutton, Surrey, UK; ^4^ The Myeloma Institute, University of Arkansas for Medical Sciences, Little Rock, Arkansas, USA; ^5^ Department of Haematology, Newcastle University, Newcastle, UK

**Keywords:** multiple myeloma, inherited risk, exome sequencing

## Abstract

The genetic basis underlying the inherited risk of developing multiple myeloma (MM) is largely unknown. To examine the impact of rare protein altering variants on the risk of developing MM we analyzed high-coverage exome sequencing data on 513 MM cases and 1,569 healthy controls, performing both single variant and gene burden tests. We did not identify any recurrent coding low-frequency alleles (1–5%) with moderate effect that were statistically associated with MM. In a gene burden analysis we did however identify a promising relationship between variation in the marrow kinetochore microtubule stromal gene *KIF18A*, which plays a role in control mitotic chromosome positioning dynamics, and risk of MM (*P* =3.6×10^−6^). Further analysis showed *KIF18A* displays a distinct pattern of expression across molecular subgroups of MM as well as being associated with patient survival. Our results inform future study design and provide a resource for contextualizing the impact of candidate MM susceptibility genes.

## INTRODUCTION

Multiple myeloma (MM) is a malignancy of plasma cells [1] for which there is an increasing incidence as the population ages. Case-control and cohort studies have consistently demonstrated a two to four-fold increased risk of MM in first-degree relatives of MM patients supporting the role of inherited susceptibility in tumour development [2].

Defining the genetic basis of this risk has proven difficult but recent genome-wide association studies (GWAS) have provided the first direct evidence for genetic susceptibility to MM, identifying risk single nucleotide polymorphisms (SNPs) at several independent loci [3–5]. Statistical modelling indicates that additional common variants with small effect should be identifiable by further GWAS. However, other types of variants should also be important and inference from studies of other cancers shows it is likely that rare, high-impact variants also contribute to the heritable risk of MM. Identifying such variants is important as this class of susceptibility can provide important insights into the molecular basis of familial and sporadic tumorigenesis. Furthermore, improved understanding of the molecular factors involved in tumorigenesis through such mechanisms has provided a basis for the rational development of targeted therapies for a number of cancers. While imputation broadens the accessible frequency spectrum of GWAS datasets, its fidelity is typically restricted to the detection of variants having minor allele frequencies >0.01. Other methodologies offer advantages over this and there is a strong rationale for searching for rare-disease associated alleles directly utilising high-throughput sequencing.

Since the exome is a highly enriched subset of the genome in which to conduct such screens, we have searched for rare high impact variants influencing MM risk by analysing whole-exome sequencing (WES) data on 553 cases of MM and 1,609 UK controls.

## RESULTS

We first examined individual recurrent variants with MAF ≤ 5% for an association with MM risk. No association was statistically significant after adjustment for multiple testing (*i.e. P* >2.02×10^−6^; Supplementary Table 1). The strongest association was observed for a synonymous variant rs13300554 in the gene *SEC16A* (hg19 chr9:g.139357939.A>G, risk allele G, MAF=0.014, OR=2.55, P=6.27×10^−5^). Relaxing criteria to include common variants the strongest association was provided by the SNP rs7188880 (hg19 chr16:g.74664810.A>T, risk allele A, MAF =0.46, OR =1.41, *P* =2.15×10^−6^), a synonymous SNP mapping to the gene encoding *RFWD3*, which was of borderline significance. rs7188880 is in strong linkage disequilibrium with the missense variant rs7193541 (*r^2^*=0.65, *D’*=0.96) previously shown by GWAS to influence MM risk [6].

We next examined the impact of rare alleles (MAF<1%) collectively within a gene on MM risk by aggregating SNVs and indels (‘T1′ test) in each gene and comparing the counts between cases and controls. Acknowledging the limitations of *in-silico* prediction to enrich for harmful alleles, we considered three nested classes of variant (see Methods): ‘disruptive’ (Class 1), ‘predicted damaging’ (Class 2) and ‘all non-synonymous’ (Class 3).

**Figure 1:**
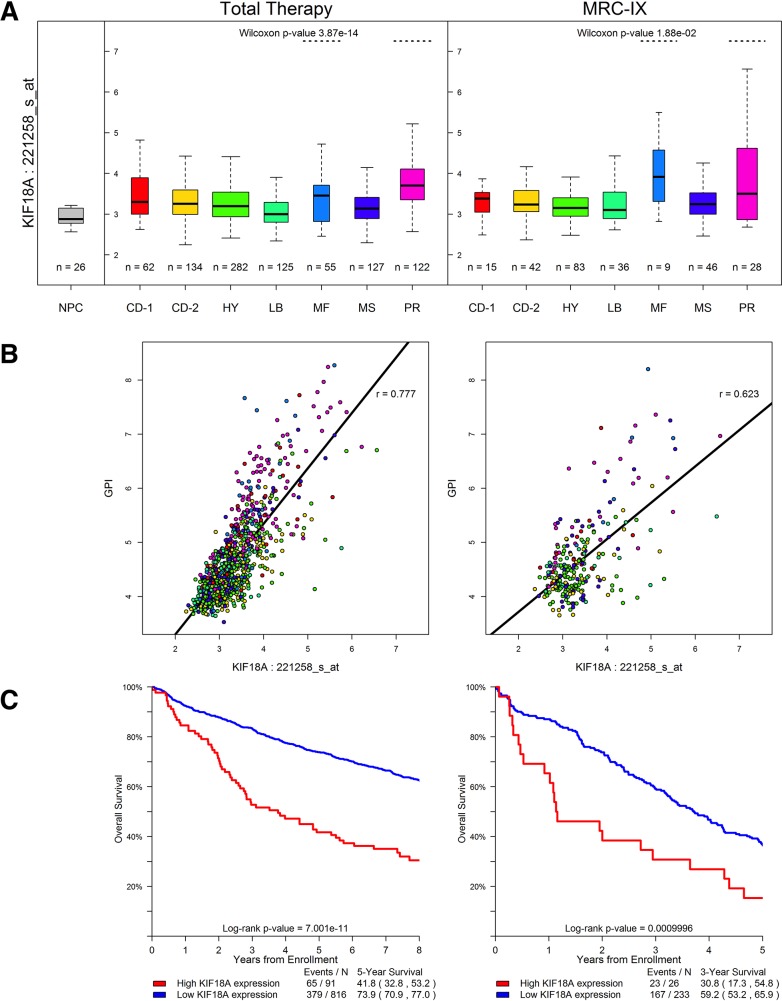
*KIF18A* expression in the Total Therapy and MRC-IX (MRC Myeloma-IX) trials **(A)** *KIF18A* expression for the seven established MM molecular subtypes [27]. TC classification groups are generated by molecular classification of patients based on unsupervised hierarchical clustering. Y-axis denotes normalized log2 *KIF18A* expression. On the boxplot the width of the boxes corresponds to the group size; the thick black line to the median; the vertical extremities of the boxes correspond to the lower and upper quartiles. The CD-1 and CD-2 groups relate to IgH translocation cases with activating *CCND1* or *CCND3*, the CD-2 group is distinguished from CD-1 by the expression of *CD20* and *PAX5*. MS group defines upregulation of *FGFR3* and/or *MMSET*, whilst the MF group is characterized by *c-MAF* or *MAFB*. LB group is defined by a low number of bone lesions. HY group that contains HD cases and PR group is characterized by the overexpression of cancer-testis antigens, cell cycle and proliferation-related genes. *KIF18A* is significantly highly expressed in subtypes MF and PR. The level of expression of *KIF18A* in normal plasma cells (NPC) is shown. **(B)** Relationship between gene expression-based proliferation index [7] (GPI) and *KIF18A* expression. **(C)** *KIF18A* expression and prognosis in MM. High expression (top 10%) of *KIF18A* is significantly associated with worse survival in the Total Therapy trial, and the MRC-IX trial data exhibits the same trend.

No individual gene showed a significant enrichment of Class 1 variation in cases, the strongest association being shown for *CC2D2B* (*P* =4.2×10^−4^; Table 1). Furthermore, there was no global over-representation of associations across Class 1 variants (*e.g*. 6 vs. 3.3 expected at *P* ≤0.01, Supplementary Figure 1C; Supplementary Table 2). While no gene was formally statistically significant across any class when adjusting for multiple tests (*i.e*. *P* >3.3×10^−6^; Table 1; Supplementary Table 2) we did identify a promising relationship with Class 3 variants in the marrow kinetochore microtubule stromal gene *KIF18A* gene (*P* =3.6×10^−6^; Supplementary Table 3 details specific variants). For 14 of the 16 cases where matched tumor WES data was available there was no statistical evidence for preferential loss of wild-type allele in carriers (*P* = 0.19, one-sided binomial test). Analyzing Total Therapy and MRC-IX trial data, *KIF18A* expression was significantly elevated in the *MAF/MAFB* (MF) and *overexpression of proliferation-related genes* (PR) subtypes (*P* =3.9×10^−14^ and *P* =0.019 in Total Therapy and MRC-IX data sets, respectively; Figure 1A). The level of expression of *KIF18A* in normal plasma cells was also found to be lower than that seen in MM (*P*<0.001; Figure 1A). *KIF18A* expression is significantly correlated with the gene expression-based proliferation index (GPI) [7] in both data sets (Figure 1B). In addition, there is a significant association between high expression of *KIF18A* (top 10% versus lower 90%) and poor outcome in the Total Therapy and MRC-IX trials (*P*<0.001 in both sets; Figure 1C). However, in a multivariate cox regression including MF and PR subgroup designation, elevated *KIF18A* expression, and GPI, *KIF18A* expression did not retain significance suggesting that proliferation is the primary independent prognostic factor. Pathway analysis revealed significantly increased activation of Cell Cycle and DNA replication pathways in samples with high expression of *KIF18A*.

**Table 1:** Summary of the gene burden results; genes are ordered by their minimum *P*-value (*P*_min_) in any of the 3 classes

Gene	*P*_min_	Class 1 variants						Class 2 variants						Class 3 variants					
		*P*	Ca.	Co.	No. uniquevariants			*P*	Ca.	Co.	No. uniquevariants			*P*	Ca.	Co.	No. uniquevariants		
					Total	Ca.	Co.				Total	Ca.	Co.				Total	Ca.	Co.
*KIF18A*	3.6 × 10^−6^	-	0	0	0	0	0	1.5 × 10^−1^	2	2	4	2	2	3.6 × 10^−6^	16	7	13	10	6
*GPRC5A*	1.1 × 10^−4^	-	0	0	0	0	0	1.1 × 10^−4^	6	0	6	6	0	6.1 × 10^−2^	18	35	13	10	6
*CNTN1*	1.8 × 10^−4^	-	0	0	0	0	0	1.7 × 10^−3^	7	3	5	4	3	1.8 × 10^−4^	13	8	15	9	8
*TMEM79*	2.8 × 10^−4^	7.2 × 10^−1^	0	2	1	0	1	7.6 × 10^−1^	2	11	7	1	7	2.8 × 10^−4^	33	42	12	4	12
*TBC1D17*	3.3 × 10^−4^	-	0	0	0	0	0	6.2 × 10^−4^	11	7	7	6	3	3.3 × 10^−4^	15	12	13	9	8
*OXA1L*	4.1 × 10^−4^	4.5 × 10^−3^	14	16	3	1	3	7.9 × 10^−4^	18	19	8	4	6	4.1 × 10^−4^	38	59	16	10	13
*CC2D2B*	4.2 × 10^−4^	4.2 × 10^−4^	8	3	8	7	1	7.1 × 10^−4^	9	5	11	8	3	4.0 × 10^−3^	10	11	17	10	7
*HSD11B2*	4.5 × 10^−4^	-	0	0	0	0	0	1.2 × 10^−1^	1	0	1	1	0	4.5 × 10^−4^	5	0	4	4	0
*ADAM29*	4.5 × 10^−4^	3.0 × 10^−2^	2	0	2	2	0	4.5 × 10^−4^	5	0	4	4	0	1.7 × 10^−2^	10	12	14	8	9
*RALGPS2*	4.7 × 10^−4^	7.5 × 10^−3^	3	0	5	5	0	4.7 × 10^−4^	6	1	9	8	1	1.3 × 10^−2^	6	7	17	10	7
*PRUNE2*	4.7 × 10^−4^	1.4 × 10^−2^	7	6	3	2	2	7.4 × 10^−3^	41	78	31	17	25	4.7 × 10^−4^	74	149	76	40	60
*ALDH1L2*	5.1 × 10^−4^	4.6 × 10^−2^	5	5	6	4	4	5.1 × 10^−4^	18	19	21	15	14	2.6 × 10^−3^	19	27	27	16	19
*ABCD4*	5.7 × 10^−4^	-	0	0	0	0	0	5.7 × 10^−4^	8	3	6	5	2	1.7 × 10^−1^	10	20	14	7	9
*CSMD2*	5.9 × 10^−4^	-	0	0	0	0	0	3.3 × 10^−2^	19	34	13	8	9	5.9 × 10^−4^	47	76	41	24	27
*SOX13*	6.2 × 10^−4^	-	0	0	0	0	0	-	0	0	0	0	0	6.2 × 10^−4^	11	7	6	5	4
*LPCAT2*	6.4 × 10^−4^	-	0	0	0	0	0	6.4 × 10^−4^	6	1	2	2	1	6.4 × 10^−2^	15	28	6	3	5
*PPY*	6.4 × 10^−4^	-	0	0	0	0	0	2.2 × 10^−3^	5	1	2	2	1	6.4 × 10^−4^	6	1	3	3	1
*ACTL6B*	6.4 × 10^−4^	-	0	0	0	0	0	-	0	0	0	0	0	6.4 × 10^−4^	7	2	2	2	1
*UNC13C*	6.5 × 10^−4^	5.7 × 10^−2^	3	3	5	5	1	6.5 × 10^−4^	30	46	26	15	16	5.5 × 10^−3^	44	84	55	23	40
*ABCA6*	6.9 × 10^−4^	1.0 × 10^−1^	4	5	7	4	4	1.8 × 10^−3^	23	31	26	12	19	6.9 × 10^−4^	35	53	43	22	31
*ABCB11*	9.9 × 10^−4^	1.2 × 10^−1^	1	0	1	1	0	4.8 × 10^−2^	8	11	15	7	9	9.9 × 10^−4^	29	43	28	13	21

Significance threshold *P* = 3.3× 10^−6^; number of cases = 513; number of controls = 1569; full results are shown in Supplementary Table 1

Ca. = Cases; Co. = Controls

Since many cancer susceptibility genes (CSGs) have pleiotropic effects, influencing the risk of different cancer types to varying degrees, we assessed a set of 114 well-established CSGs for enrichment in mutations in cases versus controls. The strongest CSGs associations were provided by *SOS1* (*P* =0.03), *BUB1B* (*P* = 0.002) and *ABCB11* (*P* =0.001) for Classes 1, 2 and 3 respectively (Supplementary Table 2). Imposing a significance threshold of 4.4×10^−4^ to address the issue of multiple testing due to evaluating the 114 CSGs, no significant association was shown.

## DISCUSSION

We report the first analysis of the contribution of rare disease-causing alleles to MM by analyzing germline WES data. Our results summarize observed variation in the largest MM germline sequencing study to date, thus providing an invaluable reference for future genetic and functional studies. We have identified *KIF18A* as a possible candidate for defining MM susceptibility. The proliferation arrest of MM cells out of niche has been shown to be associated with the widespread down regulation of mitotic and transcriptional genes [7], which includes *KIF18A*, hence variation in *KIF18A* has a strong biological basis for having a role in MM susceptibility *a priori*. Moreover elevated expression of *KIF18A* has been shown in other cancers to be associated with enhanced cell proliferation and predictive of poor prognosis [8] [9] [10].

Some regions of the genome are refractory to WES, hence we cannot exclude the possibility of disease-causing variants which could not be assayed. Accepting such a caveat a number of conclusions can be derived from our findings. First, the existence of rare recurrent protein altering variants with population frequencies of 1% or greater and conferring a RR of 4.0 seem implausible. Our analysis is however, based on UK data and does not exclude the possibility of this class of allele contributing to MM risk in populations with a more restricted allelic architecture. We acknowledge that our study had limited power to identify alleles with moderate penetrance. The use of familial cases provides a means of significantly empowering the search for rare disease-causing alleles for cancer. Although in concept an attractive strategy the number of familial MM cases are few, hence the practicality of adopting this as a means of gene identification for MM is inherently problematic. Such considerations should not however detract from performing WES or whole genome sequencing on MM families that potentially offer the prospect of discovering high-impact mutations likely to be highly informative for understanding MM biology.

## MATERIALS AND METHODS

### Patients

We analyzed WES data on 553 (307 male) patients with MM (mean age at diagnosis 65 years) that have been the subject of a previously reported study [11]. Briefly, the patients comprised 527 from the UK Medical Research Council (MRC) Myeloma XI trial and 260 from the UK MRC Myeloma IX trial. Germline DNA was extracted from EDTA venous blood samples obtained at diagnosis. Paired tumor DNA was extracted from CD138-sorted bone marrow samples where available [11]. WES of germline and tumor samples was performed using Agilent-Custom 53Mb Exome Capture (Agilent, Santa Clara, CA, USA) and Illumina HiSeq2000 technology (Illumina, San Diego, CA, USA).

### Controls

The controls comprised 1,609 healthy individuals from the UK 1958 Birth cohort [12] - 961 from the ICR1000 dataset (EGAD00001001021) [13] and an additional 648 individuals all sequenced using Illumina TruSeq 62Mb expanded exome enrichment kit in conjunction with Illumina HiSeq2000 technology.

We analyzed germline WES data on 553 UK MM patients and 1,609 1958BC controls. Cases and controls had similar sequencing metrics (Supplementary Table 4). 80 samples were excluded due to low-quality data or non-northern European ancestry leaving 513 cases and 1,569 controls for analysis.

### Variant analysis pipeline

CASAVA (v.1.8.1, Illumina) was used to extract paired end fastq files, then Stampy and BWA [14] were used to align reads to human reference genome build 37 (hg19). Alignments were processed using the Genome Analysis Tool Kit (GATK v3), according to best practices [15, 16]. Variants were called on the genomic region comprising the union of the TruSeq 62Mb capture and the Agilent-Custom 53Mb capture, plus 100bp padding at each boundary (Supplementary Table 5). Variants were called simultaneously across all case and control samples. For the loss of heterozygosity analysis, MM germline and tumor sample variants were called using Platypus [17]. The Variant Effect Predictor (VEP) was used to annotate each variant with its effect on canonical protein transcripts [18]. For the gene burden analysis if a variant received multiple relevant VEP annotations for a gene, we used only the single annotation deemed likely to have the most profound impact adopting the hierarchy: stop gained, frameshift, splice acceptor/donor variants, in-frame insertion/deletion, and lastly missense, which were additionally annotated with predicted pathogenicity using the CONDEL algorithm [19]. All variants were annotated with their distance from simple repeats, and their 100mer alignability, using the UCSC browser [20]. ClinVar was used to check variants in promising genes for previously documented evidence of pathogenicity [21]. Linkage disequilibrium (LD) between variants was retrieved using the SNAP pairwise LD online tool with SNP data set ‘1000 Genomes Pilot 1′ and population panel ‘CEU’ [22].

### Sample quality control

Germline samples were excluded for the following reasons (Supplementary Table 6): (i) sex discrepancy (n =13, using PLINK); (ii) high missingness rate (n =43, >3 standard deviations (SDs) above the mean, calculated across a set of 6,100 SNVs catalogued by dbSNPv138, hereafter called ‘*Common_SNV_Set’*); (iii) high rate of heterozygosity (n =6, >3 SDs above mean, across *Common_SNV_Set*); (iv) non-Northern European ancestry (n =16, as assessed by principal component analysis using EIGENSTRAT with HapMap Project data as reference, Supplementary Figure 2); (v) sample duplication (n =2) [23].

### Variant quality control

A variant was only considered to be present if the GATK genotype-quality was ≥30, the alternate depth was >3, and it was in an acceptable truth tranche (*i.e*. <99.5 for SNVs and <99 for indels) as per GATK best practices.

### Variant quality control for single variant analysis

For the single-variant analysis criteria were chosen to ensure the genomic inflation factor over the highest 90% of passing *P*-values remained close to unity (Supplementary Figure 1A). Variants were thus discarded if: (i) UCSC alignability ≠ 1 (100bp window size); (ii) variant within 10 bps of a simple repeat region; (iii) highly significant deviation from Hardy-Weinberg equilibrium (HWE) in cases or controls (*P* <10^−5^); (iv) no call rate significantly different between case-samples and control-samples (*P* <10^−5^); (v) no call rate across either case or control samples >0.03. Furthermore for each variant the ‘minimum possible’ *P*-value it could obtain was calculated based upon the number of variant alleles observed, by assuming all variant alleles were in cases. Variants with high minimum possible *P*-values were disregarded if this value was greater than the Bonferroni corrected significance threshold resulting from their inclusion leaving 24,752 for analysis.

### Variant quality control for gene centric analysis

For the gene-centric analysis (sample, variant)-pairs were excluded if a heterozygous site was called yet the read counts in support of the reference and alternate alleles were unbalanced (*P* <0.0001, χ^2^ test). Variants were excluded across all samples if the UCSC alignability ≠ 1, the variant mapped within a simple repeat, the HWE *P*-value across cases and controls was <10^−8^, or the no call rate across either case- or control-samples was ≥25%.

### Publicly accessible control data

As additional sources of variant frequency in controls we referenced UK10K exome data (ALSPAC n =1,828 and TWINSUK n =1,754) and Exome Aggregation Consortium (ExAC– release 0.3, non-Finnish European population, excluding samples analyzed by The Cancer Genome Atlas).

### Statistical analyses

#### Single variant association

The difference in allele frequency in cases and controls was assessed using Fisher's exact test (two-sided) implemented in R [24]. A *P*-value of <2.02×10^−6^ was declared as significant; corresponding to a Bonferroni correction for 24,752 tests.

### Gene-centric analysis

To test whether rare mutations contribute to MM we performed a collapsing burden test imposing a maximal MAF threshold of 1% (T1 test). We applied the T1 test for three nested classes of variant: Class 1) ‘disruptive’ (nonsense, frameshift); Class 2) ‘predicted damaging’ (disruptive + missense predicted to be damaging by CONDEL, and splice site acceptor/donors); Class 3) ‘all non-synonymous’ (predicted damaging + all sufficiently rare non-synonymous variants). To ensure nominal power to identify associations we restricted our analysis to genes featuring variants in ≥10 samples amongst cases and controls (Supplementary Figure 3). Exome-wide significance was considered to be *P* =3.3×10^−6^, corresponding to a Bonferroni correction for the 15,358 tests conducted when considering all classes. Significance levels were assessed using a one-sided permutation test on case/control status.

### Study power

Single variants were characterized by their minor allele frequency (MAF) and relative risk (RR); for (MAF, RR)-pairs discovery power was analysed by simulating 10,000 draws of case and control alleles from the population (Supplementary Figure 4A). Gene based analysis was treated in a similar manner to single-variants, except instead of using MAF, the “% of population with a Class 1 (or 2 or 3) variant in the gene-of-interest” was used (Supplementary Figure 4B).

### Loss of heterozygosity

A search for loss of heterozygosity in paired tumor samples was performed using ExomeCNV (v1.4) [25].

### Impact of pleiotropic effects of cancer susceptibility genes

In addition to performing an agnostic search for novel susceptibility genes, under the hypothesis that some MM cases might be ascribable to the pleotropic effects of known cancer susceptibility genes (CSG) we also adopted a focused assessment of the 114 established CSGs [26].

### Expression analysis of *KIF18A*

The impact of *KIF18A* (221258_s_at) expression in MM was from Affymetrix Human Genome U133 2.0 Plus array data in plasma cells from Total therapy (GSE2658) and MRC Myeloma IX trial patients (NCBI GEO Datasets GSE31161) by TC classification. Differences in *KIF18A* expression between MM subtypes was assessed using the Wilcoxon test. Significance of difference in patient survivorship was determined using the log-rank test with “high” expression defined as the top 10% of samples, and “low” as the bottom 90%. All statistical analyses were performed using R version 3.2.1 software. A *P*-value of 0.05 (two-sided) was considered to be statistically significant.

### Data availability

Whole-exome sequence data that support the findings of this study have been deposited in EGA with accession codes EGAS00001001 and EGAD00001001021. Expression data that support the findings of this study have been deposited in GEO with accession codes GSE2658 and GSE31161. The remaining data are contained within the paper and Supplementary Files or available from the author upon request.

### Web addresses

Genome Analysis Tool Kit (GATKv3): https://www.broadinstitute.org/gatk

ClinVar: http://www.ncbi.nlm.nih.gov/clinvar

Uk10k: http://www.uk10k.org

Exome Variant Server, NHLBI GO Exome Sequencing Project (ESP), Seattle, WA: http://evs.gs.washington.edu/EVS

PLATYPUS: http://www.well.ox.ac.uk/platypus

EXAC: http://exac.broadinstitute.org/

PLINK: http://pngu.mgh.harvard.edu/~purcell/plink/

SNAP: https://www.broadinstitute.org/mpg/snap/ldsearchpw.php

## SUPPLEMENTARY MATERIALS FIGURES AND TABLES















## References

[1] Kyle RA, Rajkumar SV (2004). Multiple myeloma. N Engl J Med.

[2] Altieri A, Chen B, Bermejo JL, Castro F, Hemminki K (2006). Familial risks and temporal incidence trends of multiple myeloma. Eur J Cancer.

[3] Broderick P, Chubb D, Johnson DC, Weinhold N, Forsti A, Lloyd A, Olver B, Ma YP, Dobbins SE, Walker BA, Davies FE, Gregory WA, Child JA (2012). Common variation at 3p22.1 and 7p15.3 influences multiple myeloma risk. Nat Genet.

[4] Chubb D, Weinhold N, Broderick P, Chen B, Johnson DC, Forsti A, Vijayakrishnan J, Migliorini G, Dobbins SE, Holroyd A, Hose D, Walker BA, Davies FE (2013). Common variation at 3q26.2, 6p21.33, 17p11.2 and 22q13.1 influences multiple myeloma risk. Nat Genet.

[5] Weinhold N, Johnson DC, Chubb D, Chen B, Forsti A, Hosking FJ, Broderick P, Ma YP, Dobbins SE, Hose D, Walker BA, Davies FE, Kaiser MF (2013). The CCND1 c.870G>A polymorphism is a risk factor for t(11;14)(q13;q32) multiple myeloma. Nat Genet.

[6] Mitchell JS, Li N, Weinhold N, Forsti A, Ali M, van Duin M, Thorleifsson G, Johnson DC, Chen B, Halvarsson BM, Gudbjartsson DF, Kuiper R, Stephens OW (2016). Genome-wide association study identifies multiple susceptibility loci for multiple myeloma. Nat Commun.

[7] Hose D, Reme T, Hielscher T, Moreaux J, Messner T, Seckinger A, Benner A, Shaughnessy JD, Barlogie B, Zhou Y, Hillengass J, Bertsch U, Neben K (2011). Proliferation is a central independent prognostic factor and target for personalized and risk-adapted treatment in multiple myeloma. Haematologica.

[8] Wang L, Yang S, Sun R, Lu M, Wu Y, Li Y (2016). [Expression of KIF18A in gastric cancer and its association with prognosis]. Zhonghua Wei Chang Wai Ke Za Zhi.

[9] Chen QI, Cao B, Nan N, Wang YU, Zhai XU, Li Y, Chong T (2016). Elevated expression of KIF18A enhances cell proliferation and predicts poor survival in human clear cell renal carcinoma. Exp Ther Med.

[10] Liao W, Huang G, Liao Y, Yang J, Chen Q, Xiao S, Jin J, He S, Wang C (2014). High KIF18A expression correlates with unfavorable prognosis in primary hepatocellular carcinoma. Oncotarget.

[11] Walker BA, Boyle EM, Wardell CP, Murison A, Begum DB, Dahir NM, Proszek PZ, Johnson DC, Kaiser MF, Melchor L, Aronson LI, Scales M, Pawlyn C (2015). Mutational Spectrum, Copy Number Changes, and Outcome: Results of a Sequencing Study of Patients With Newly Diagnosed Myeloma. J Clin Oncol.

[12] Power C, Elliott J (2006). Cohort profile: 1958 British birth cohort (National Child Development Study). Int J Epidemiol.

[13] Ruark E, Munz M, Renwick A, Clarke M, Ramsay E, Hanks S, Mahamdallie S, Elliott A, Seal S, Strydom A, Gerton L, Rahman N (2015). The ICR1000 UK exome series: a resource of gene variation in an outbred population. F1000Res.

[14] Lunter G, Goodson M (2011). Stampy: a statistical algorithm for sensitive and fast mapping of Illumina sequence reads. Genome Res.

[15] McKenna A, Hanna M, Banks E, Sivachenko A, Cibulskis K, Kernytsky A, Garimella K, Altshuler D, Gabriel S, Daly M, DePristo MA (2010). The Genome Analysis Toolkit: a MapReduce framework for analyzing next-generation DNA sequencing data. Genome Res.

[16] DePristo MA, Banks E, Poplin R, Garimella KV, Maguire JR, Hartl C, Philippakis AA, del Angel G, Rivas MA, Hanna M, McKenna A, Fennell TJ, Kernytsky AM (2011). A framework for variation discovery and genotyping using next-generation DNA sequencing data. Nat Genet.

[17] Rimmer A, Phan H, Mathieson I, Iqbal Z, Twigg SR, Consortium WGS, Wilkie AO, McVean G, Lunter G (2014). Integrating mapping-, assembly- and haplotype-based approaches for calling variants in clinical sequencing applications. Nat Genet.

[18] McLaren W, Pritchard B, Rios D, Chen Y, Flicek P, Cunningham F (2010). Deriving the consequences of genomic variants with the Ensembl API and SNP Effect Predictor. Bioinformatics.

[19] Gonzalez-Perez A, Lopez-Bigas N (2011). Improving the assessment of the outcome of nonsynonymous SNVs with a consensus deleteriousness score. Condel. Am J Hum Genet.

[20] Kent WJ, Sugnet CW, Furey TS, Roskin KM, Pringle TH, Zahler AM, Haussler D (2002). The human genome browser at UCSC. Genome Res.

[21] Landrum MJ, Lee JM, Riley GR, Jang W, Rubinstein WS, Church DM, Maglott DR (2014). ClinVar: public archive of relationships among sequence variation and human phenotype. Nucleic Acids Res.

[22] Johnson AD, Handsaker RE, Pulit SL, Nizzari MM, O'Donnell CJ, de Bakker PI (2008). SNAP: a web-based tool for identification and annotation of proxy SNPs using HapMap. Bioinformatics.

[23] Price AL, Patterson NJ, Plenge RM, Weinblatt ME, Shadick NA, Reich D (2006). Principal components analysis corrects for stratification in genome-wide association studies. Nat Genet.

[24] Team RC (2015). R: A language and environment for statistical computing. R Foundation for Statistical Computing.

[25] Sathirapongsasuti JF, Lee H, Horst BA, Brunner G, Cochran AJ, Binder S, Quackenbush J, Nelson SF (2011). Exome sequencing-based copy-number variation and loss of heterozygosity detection. ExomeCNV. Bioinformatics.

[26] Rahman N (2014). Realizing the promise of cancer predisposition genes. Nature.

[27] Zhan F, Huang Y, Colla S, Stewart JP, Hanamura I, Gupta S, Epstein J, Yaccoby S, Sawyer J, Burington B, Anaissie E, Hollmig K, Pineda-Roman M (2006). The molecular classification of multiple myeloma. Blood.

