# Supplementary material for: Search for rare protein altering variants influencing susceptibility to multiple myeloma
Source: Oncotarget. 2017 Mar 3;8(22):36203–10. doi: 10.18632/oncotarget.15874 (PMC5482649; doi:10.18632/oncotarget.15874)
Supplement: Supplementary file 1 [file oncotarget-08-36203-s001.pdf]

Search for rare protein altering variants influencing susceptibility to multiple myeloma

SUPPLEMENTARY TABLES, FIGURES AND LEGENDS

Supplementary Table 1: Results of the single-variant analysis; ordered by *P* value; significance threshold = 2.02×10^{-6}; number of cases = 513; number of controls = 1569

See Supplementary File 1

Supplementary Table 2: Full table of the gene burden results; genes are ordered by their minimum *P* value in any of the 3 classes; significance threshold *P* =3.3×10^{-6}; number of cases = 513; number of controls = 1569

See Supplementary File 2

Supplementary Table 3: Details of the burden of rare Class 3 variation in *KIF18A*

See Supplementary File 3

Supplementary Table 4: Sequencing metrics for case- and control-samples; calculated after sample quality control and gene centric variant quality control

See Supplementary File 4

Supplementary Table 5: Comparison of the exome capture regions

See Supplementary File 5

Supplementary Table 6: Number of samples excluded at each sample QC step

See Supplementary File 6

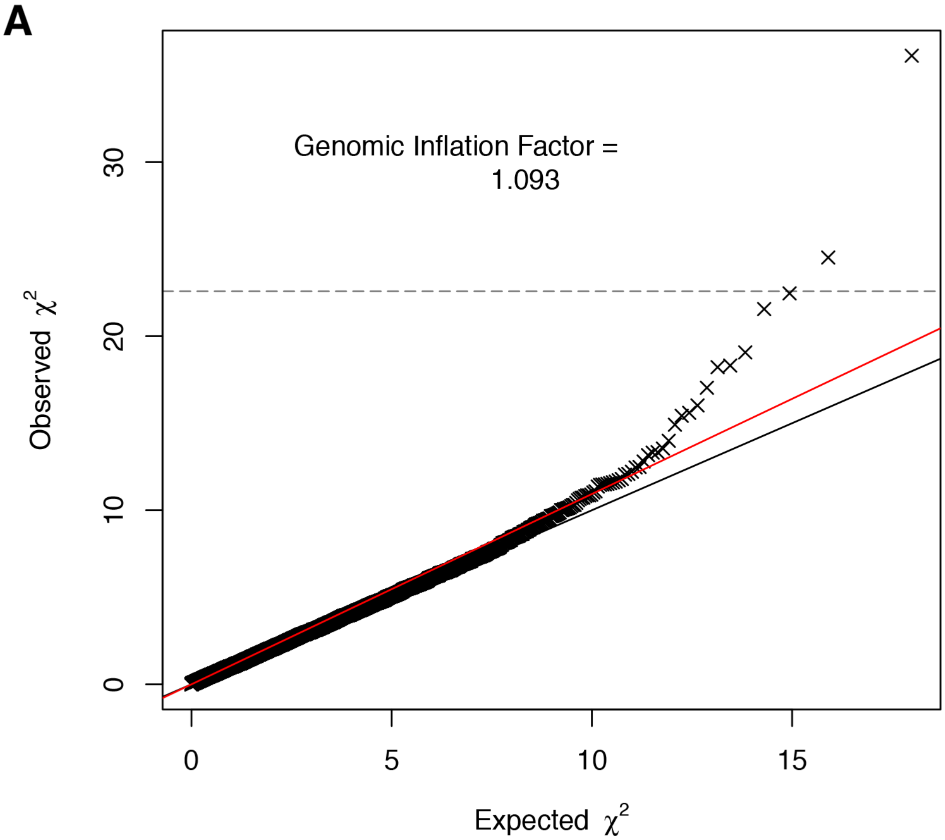

Supplementary Figure 1A: Quantile-quantile plot representing all single-variant *P* values (n =24,752); *P* values from the Fisher's exact test were mapped to χ^2 test statistic values for the purpose of calculating the genomic inflation factor; the horizontal line corresponds to the significance threshold of *P* =2.02 x 10^{-6}; the two most significant variants were excluded as their ExAC frequency did not match our control MAF.

(*Continued*)

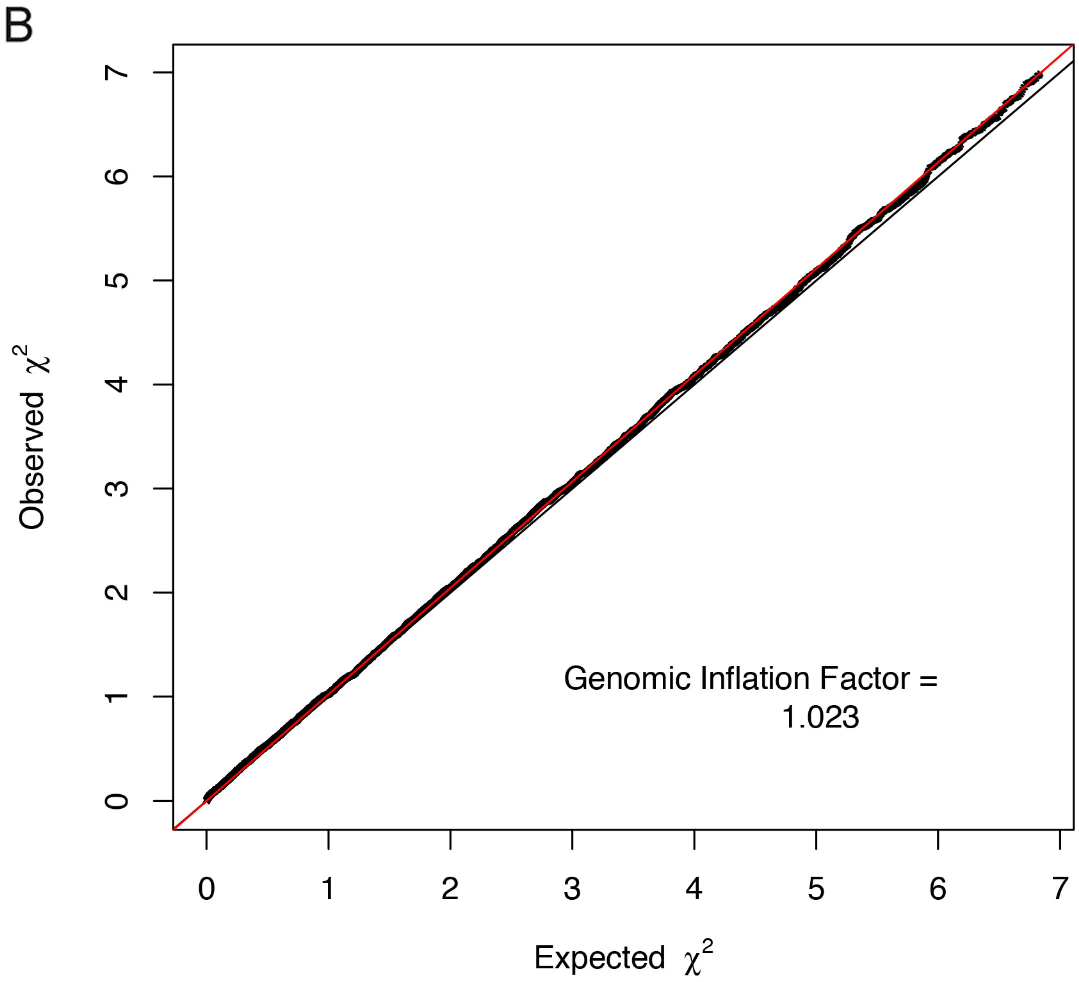

Supplementary Figure 1B (*Continued*): Quantile-quantile plot representing the least extreme 90% of single-variant *P* values; *P* values from the Fisher's exact test were mapped to χ^2 test statistic values for the purpose of calculating the genomic inflation factor.

(*Continued*)

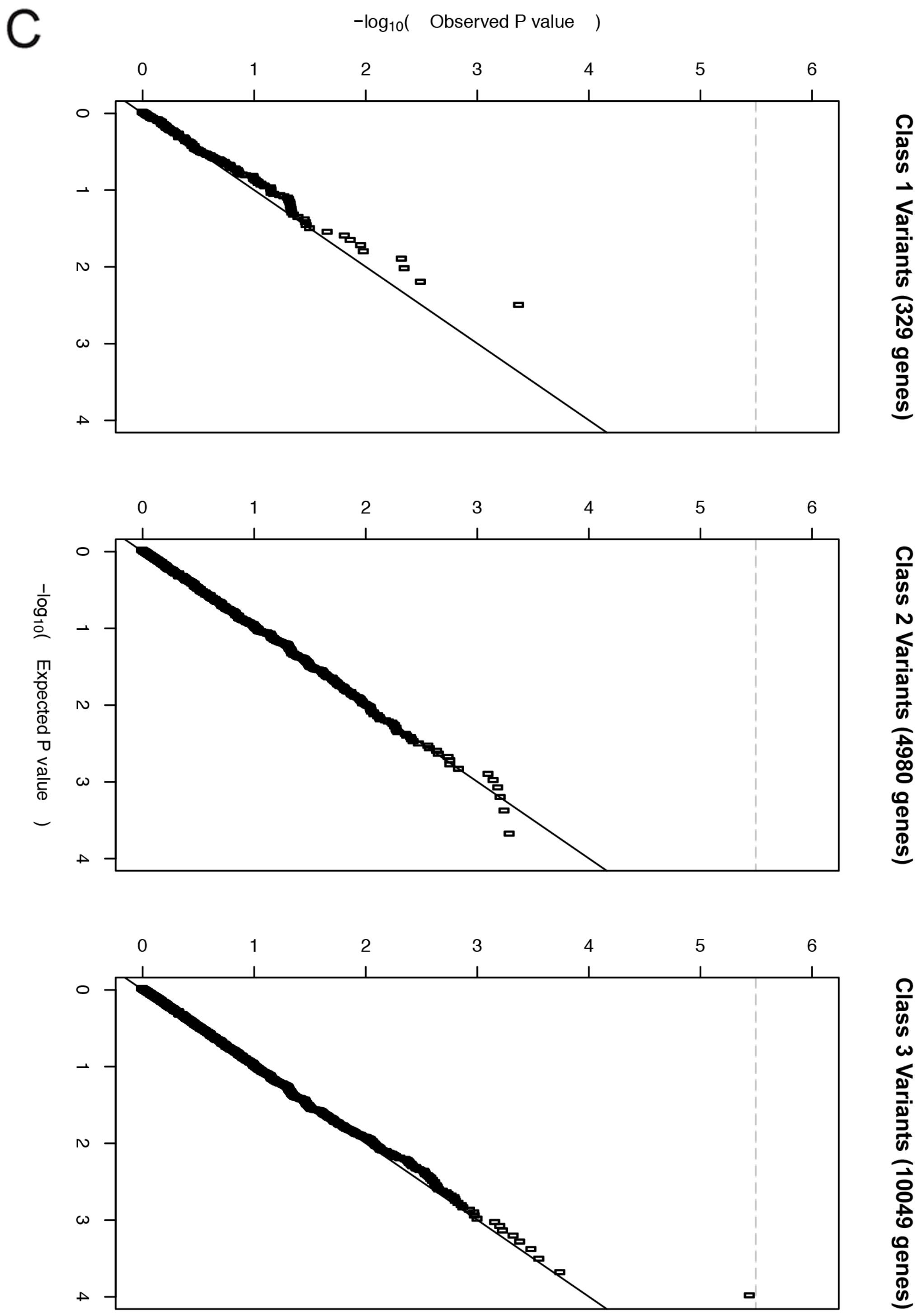

Supplementary Figure 1C (*Continued*): Quantile-quantile plots comparing the *P* values from each of the three versions of the T1 gene burden test to the expected *P* value; the solid black line is y =x; the checked vertical line corresponds to the Bonferroni corrected significance threshold of *P* =3.3 × 10^{-6}.

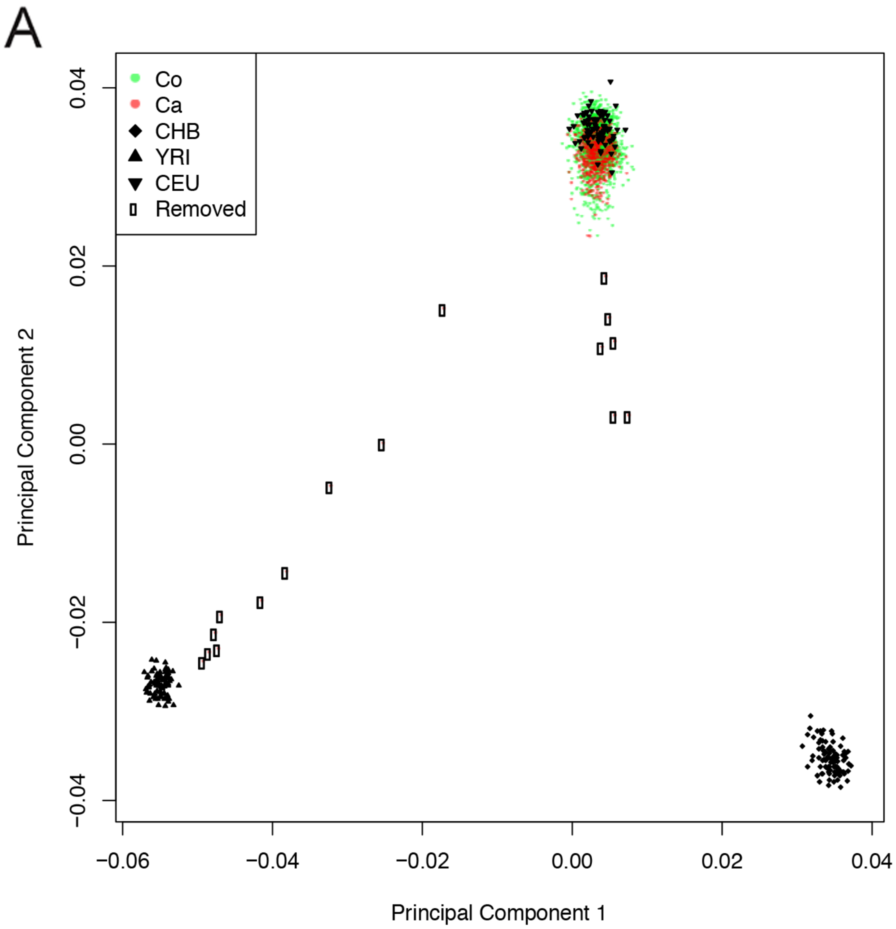

Supplementary Figure 2A: Plot of the first two principal components for each sample resulting from EIGENSTRAT analysis; case and control samples were projected on to the principal components generated using the HapMap populations; samples that are circled were discarded from analysis.

(*Continued*)

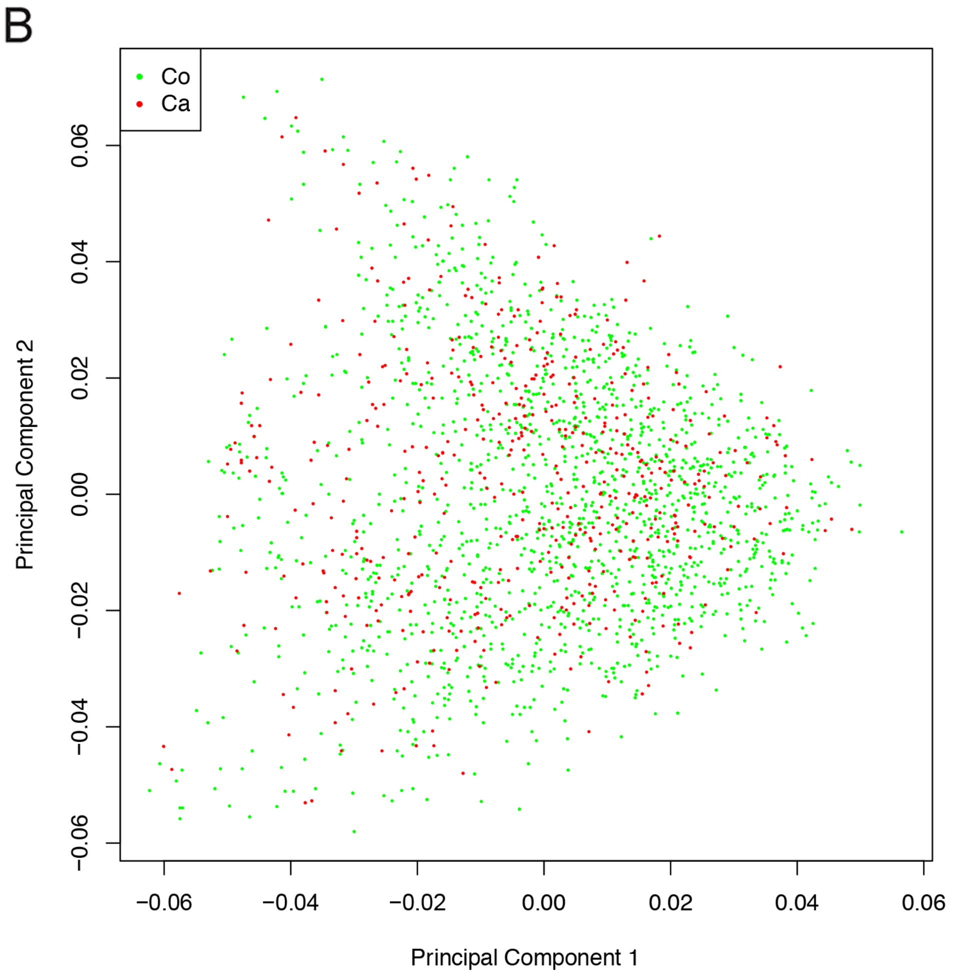

Supplementary Figure 2B (*Continued*): Plot of the first two principal components resulting from EIGENSTRAT analysis of each case and control sample that was retained for analysis; only non-outlier case and control samples were used to generate the principal components.

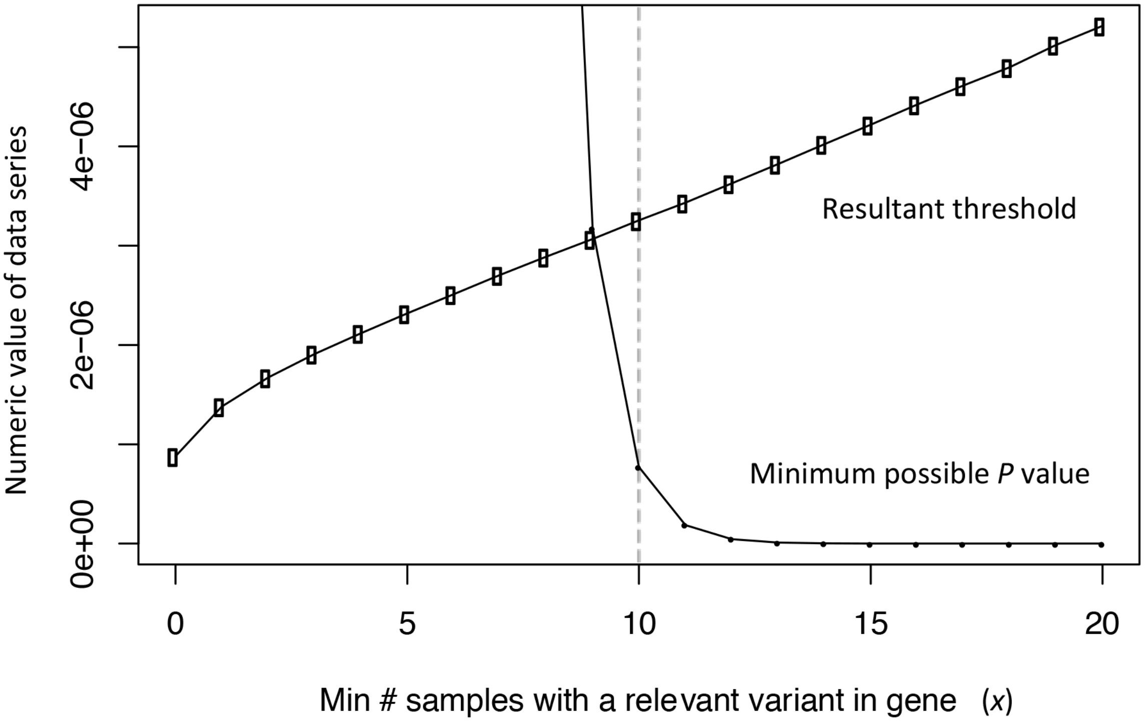

Supplementary Figure 3: For the gene burden analysis the minimum number of affected samples (across cases or controls) was set by picking the smallest number for which the minimum possible *P* value (assume all *x* affected samples are cases, not controls) was smaller than the resultant significance threshold (i.e. 0.05 / number of gene-tests with at least *x* affected samples.

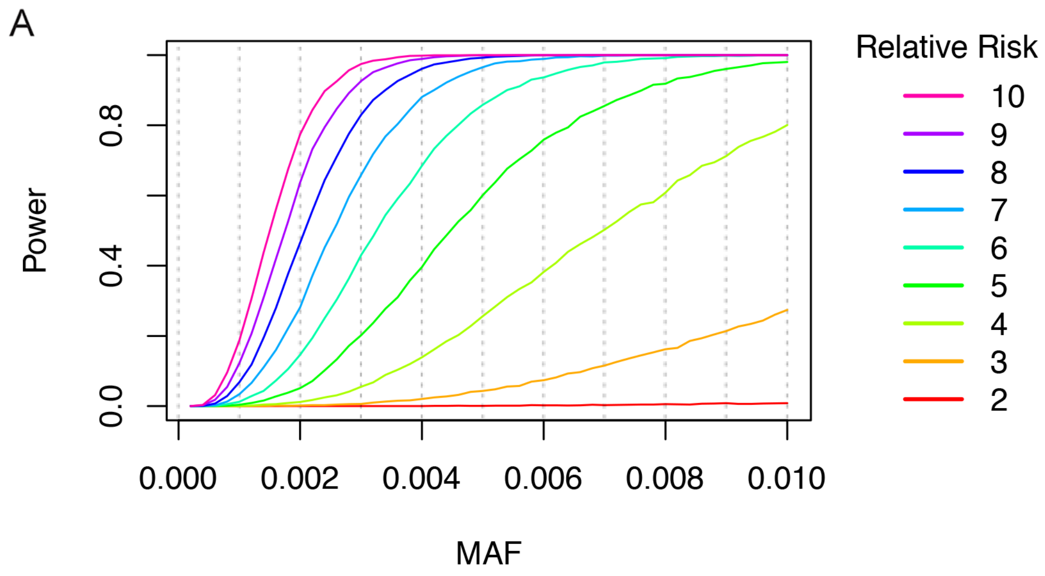

Supplementary Figure 4A: Power for single-variant analysis as a function of MAF and relative risk; power calculated by undertaking 10,000 simulated draws of cases and control alleles, and conducting a two sided Fisher's exact test on the allele counts; significance is assigned based upon a Bonferroni corrected 0.05 significance threshold.

(*Continued*)

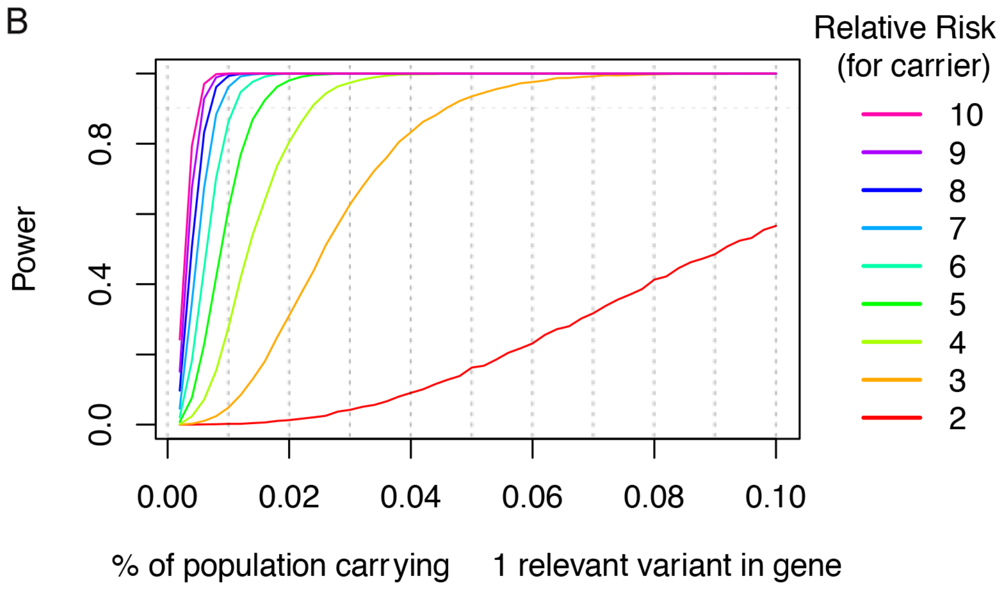

Supplementary Figure 4B (*Continued*): Power for gene-burden analysis, as a function of the percentage of the population who carry a relevant variant (Class 1, 2 or 3) in the gene-of-interest, and the relative risk conferred to carriers of a relevant variant in the gene-of-interest; power calculated by undertaking 10,000 simulated draws of cases and control individuals, and conducting a one sided Fisher's exact test on the counts; significance is assigned based upon a Bonferroni corrected 0.05 significance threshold.
