# Supplementary material for: Search for rare protein altering variants influencing susceptibility to multiple myeloma
Source: Oncotarget. 2017 Mar 3;8(22):36203–10. doi: 10.18632/oncotarget.15874 (PMC5482649; doi:10.18632/oncotarget.15874)
Supplement: Supplementary file 5 [file oncotarget-08-36203-s005.docx]

Supplementary Table S4: sequencing metrics for case- and control-samples; calculated after sample quality control and gene centric variant quality control

|  | **Controls** | **Cases** |  |  | |  |
| --- | --- | --- | --- | --- | --- | --- |
|  |  |  |  |  | |  |
| Exome Capture Kit | TruSeq 62Mb | Agilent-Custom 53Mb | | | | |
| Median % capture region covered >20x | 77% | 85% |  | |  | |
| homo_alt | 19,819 | 21,395 |  | |  | Mean counts for VEP variant types |
| het | 35,416 | 36,852 |  | |  |  |
| snp | 50,937 | 53,730 |  | |  |  |
| indel | 4,298 | 4,517 |  | |  |  |
| snp_in_dbsnp | 50,191 | 52,957 |  | |  |  |
| snp_wt_rsid | 50,160 | 5900 |  | |  |  |
| indel_in_dbsnp | 2,983 | 3,178 |  | |  |  |
| indel_wt_rsid | 3,399 | 3,599 |  | |  |  |
| splice_acceptor_variant | 20 | 20 |  | |  |  |
| splice_donor_variant | 8 | 18 |  | |  |  |
| stop_gained | 40 | 40 |  | |  |  |
| frameshift_variant | 101 | 108 |  | |  |  |
| stop_lost | 11 | 11 |  | |  |  |
| initiator_codon_variant | 7 | 6 |  | |  |  |
| inframe_insertion | 58 | 62 |  | |  |  |
| inframe_deletion | 56 | 58 |  | |  |  |
| missense_variant | 7,085 | 7,223 |  | |  |  |
| splice_region_variant | 1,672 | 1,721 |  | |  |  |
| incomplete_terminal_codon_variant | 0 | 0 |  | |  |  |
| stop_retained_variant | 6 | 6 |  | |  |  |
| synonymous_variant | 8,361 | 8,575 |  | |  |  |
| coding_sequence_variant | 11 | 12 |  | |  |  |
| mature_miRNA_variant | 2 | 3 |  | |  |  |
| X5_prime_UTR_variant | 935 | 865 |  | |  |  |
| X3_prime_UTR_variant | 2,502 | 2,101 |  | |  |  |
| non_coding_transcript_exon_variant | 1,154 | 1,121 |  | |  |  |
| intron_variant | 20,663 | 23,307 |  | |  |  |
| NMD_transcript_variant | 0 | 0 |  | |  |  |
| non_coding_transcript_variant | 3 | 4 |  | |  |  |
| upstream_gene_variant | 5,858 | 6,053 |  | |  |  |
| downstream_gene_variant | 6,671 | 6,933 |  | |  |  |
